# High Polygenic Risk of CAD Is Similar to That of Diabetes or Ischemic Stroke

**DOI:** 10.1016/j.jacadv.2026.103156

**Published:** 2026-08-05

**Authors:** Rami Hammoura, Mikael Åkerlund, Linda Holmquist Mengelbier, Olle Melander

**Affiliations:** a Department of Clinical Sciences, Lund University, Malmö, Sweden; b Skåne University Hospital, Department of Emergency and Internal Medicine, Malmö, Sweden

**Keywords:** genome-wide polygenic score, polygenetic risk of coronary artery disease, prediction of cardiovascular disease

**What is the clinical question addressed?**

Does top 20% of genome-wide polygenic score identify coronary artery disease risk equal to that of diabetes mellitus and ischemic stroke, independently of traditional risk factors?

**What is the main finding?**

Top 20% of genome-wide polygenic score independently predicts coronary artery disease with similar relative and absolute risk as diabetes mellitus and ischemic stroke

Over the past years there has been vast development of genetic risk scores for prediction of a first coronary artery disease (CAD) event.^1^^,^^2^ Recently a “genome-wide polygenic score (GPS_mult_)” for CAD was developed based on genome-wide association data from >269,000 CAD cases and >1,178,000 controls (GPS_Mult_), and was shown to outperform previous polygenic CAD scores in terms of CAD prediction.^2^ In the UK Biobank, 21% of the population with highest GPS_Mult_ had a cumulative CAD incidence that was equal to that of patients with diabetes mellitus, that is, a group of patients in whom pharmacologic primary prevention with cholesterol-lowering agents is widely recommended due to high absolute risk.^2^ Interestingly, in previous randomized controlled trials of cholesterol-lowering therapy, individuals with high (top 20%) compared to low (bottom 20%) polygenetic CAD risk have consistently been shown to have about 3 times greater benefit in terms of absolute risk reduction for hard cardiovascular events both in the primary and secondary prevention settings.^3^

Here, we tested the clinical utility of GPS_Mult_ in a population without CAD at baseline outside the UK Biobank and pharmacologic randomized controlled trials, that is, in a large Swedish population by: 1) evaluating its ability to predict first incident CAD events independently of traditional risk factors (TRFs) and first degree family history of myocardial infarction (FH + MI); and 2) comparing the relative and absolute CAD risk in people with high (top 20%) GPS_Mult_ with that of patients with diabetes or ischemic stroke, that is, patient groups in whom cholesterol-lowering pharmacologic therapy is currently widely recommended.

The population-based prospective cohort study Malmö Diet and Cancer (MDC), in whom baseline risk factors were measured and blood for DNA extraction was biobanked 1991 to 1996, underwent Illumina array-based genome wide genotyping and imputation with Haplotype Reference Consortium (HRC) as reference panel. GPS_Mult_^2^ was calculated in 29,004 successfully genotyped MDC participants.^2^ The top quintile of GPS_Mult_ was defined as high genetic risk and compared with the remaining part of the population (quintiles 1-4 of GPS_Mult_). After exclusion of 716 individuals with CAD and 596 with lipid-lowering medication before the baseline exam as well as another 2,289 individuals with missing data on any of the risk factors used in multivariate analyses, 25,403 individuals with complete data were available for analyses. We used record linkage to the Swedish national inpatient, cause of death, and SwedeHeart registries to retrieve CAD events (fatal or nonfatal MI, death due to coronary heart disease, or revascularization with coronary artery bypass grafting or percutaneous coronary intervention) during follow-up as described previously.^4^ Cox proportional hazards model was used to relate GPS_Mult_ to CAD incidence in models adjusted for age and sex (model 1), age, sex and FH + MI (model 2) and additional adjustment for smoking, systolic blood pressure, antihypertensive medication, apolipoprotein-B, apolipoprotein-A1, body mass index, prevalent diabetes, and prevalent ischemic stroke (model 3). The proportionality of hazards assumption was met.

The study was approved by the Swedish Board of Ethics “2023-00503-01”.

The baseline risk factor distribution of MDC has been reported before.^4^ Briefly, the mean age was 58 years, 62% were females, 28% current smokers, and 4% had diabetes. The continuous GPS_Mult_ correlated significantly with all model 3 covariates but the r2-values in Pearson correlations (proportion of variance explained) were low, ranging from 0.001 to 0.002 for age, sex, smoking, prevalent ischemic stroke and prevalent diabetes mellitus to 0.006 to 0.009 for systolic blood pressure, ApoA1, body mass index, and family history for MI, and to 0.03 for ApoB. During a mean follow-up time of 21 years, 3,924 first CAD events occurred. High genetic risk (top 20% vs remaining 80% of GPS_Mult_) conferred a HR (95% CI) for CAD of 2.34 (2.20-2.52), *P* = 7 × 10^-136^ in model 1. After adjustment for FH + MI (model 2), which conferred a HR of 1.37 (1.29-1.46), *P* = 3 × 10^-22^, the HR for CAD in high GPS_Mult_ remained unchanged (2.30 [2.15-2.46]; *P* = 4 × 10^-128^). Additional adjustment for all CAD risk factors (model 3), which were all significant, resulted in a HR in high GPS_Mult_ of 2.0 (1.86-2.13), *P* = 2 × 10^-85^. The HR for CAD conferred by high GPS_Mult_ was similar as that conferred by diabetes at baseline (1.84 [1.63-2.07]; *P* = 4 × 10^-30^) and greater than that conferred by ischemic stroke before baseline (1.38 [1.03-1.86]; *P* = 0.03) in model 3. The model 3 adjusted cumulative incidence of CAD (absolute risk) over the entire follow-up was 28% among 4,785 individuals with high GPS_Mult_, 30% among 1,001 patients with diabetes and 23% among 167 patients with ischemic stroke (Figure 1). For CAD incidence discrimination, Harrell C index increased from 0.70 for age and sex to between 0.71 to 0.72 for addition of each of the additional individual TRFs and to 0.74 (0.76-0.78) with addition of continuous GPS_mult_. For all TRFs Harrell C index was 0.75 (0.74-0.76) which increased to 0.77 (0.76-0.78) with addition of GPS_mult_. Continuous net reclassification improvement was 0.57 (0.18-0.94) when GPS_mult_ was added on top of all TRFs.

**Figure 1:**
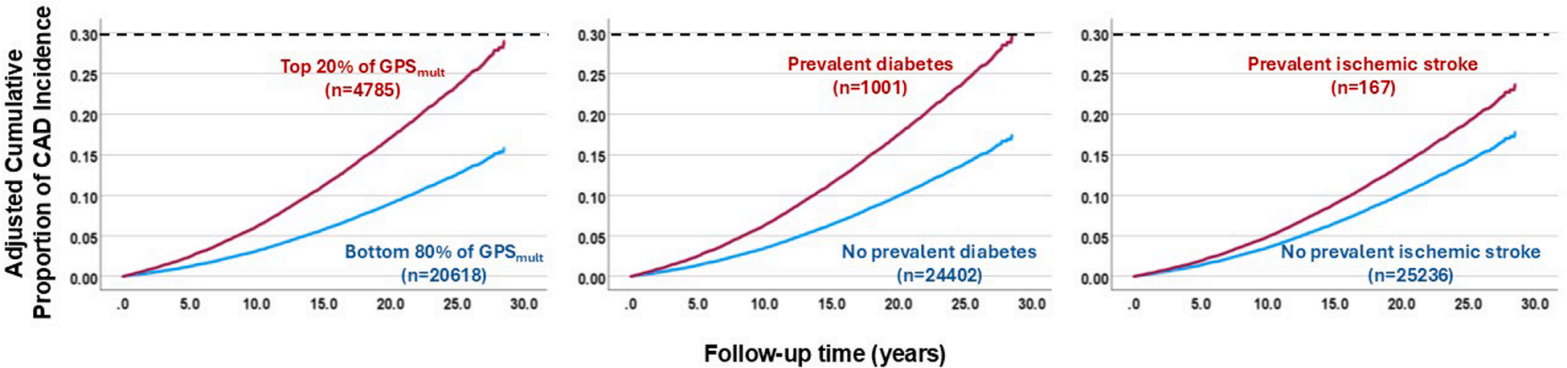
**Cumulative Model 3 Adjusted Proportion of CAD Incidence** CAD = coronary artery disease; GPS_mult_ = genome-wide polygenic score.

In summary, high polygenetic risk (top 20% of GPS_Mult_) identifies CAD-free individuals in the population independently of FH + MI and TRFs with the same relative and absolute risks of future CAD development as in patients with diabetes or ischemic stroke. In MDC, we do not have data on coronary artery calcium, another strong CAD risk enhancer.^5^ However, the stability of the genome from birth points at the primary value of GPS_Mult_ in younger ages (ie, before presence of coronary calcium becomes common). In conclusion, our results suggest that high GPS_Mult_ improves prediction of the first CAD event, which may have implications for decisions on pharmacologic primary prevention.

## Funding support and author disclosures

Funding for the study was obtained from the Swedish Scientific Council (2023-01989), the Swedish Heart and Lung Foundation (20240352), ALF-grant, and Region Skåne. The authors have reported that they have no relationships relevant to the contents of this paper to disclose.
